# Supplementary material for: Integrins are required for synchronous ommatidial rotation in the Drosophila eye linking planar cell polarity signalling to the extracellular matrix
Source: Open Biol. 2019 Aug 14;9(8):190148. doi: 10.1098/rsob.190148 (PMC6731590; doi:10.1098/rsob.190148)
Supplement: Supplemental Data [file rsob190148supp1.pdf]

Supplemental Data

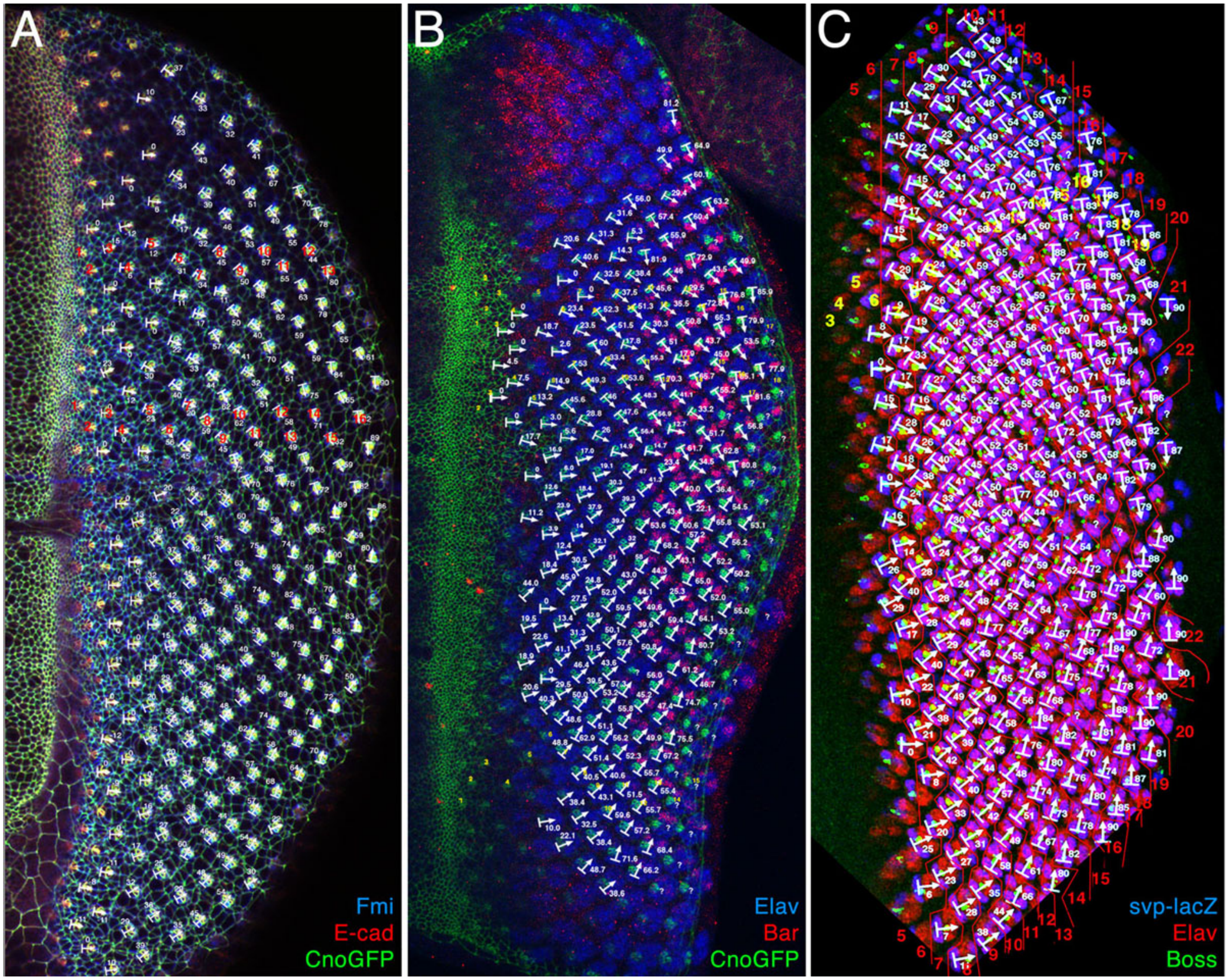

Figure S1: Rotation angle evaluation in wild-type eye discs
Examples of wild-type third instar eye discs and the respective quantifications of rotation angles. The discs are stained with markers as indicated. Individual angle values are indicated in white and columns are numbered in red in panel A and in yellow in panel C. Anterior is to the left and dorsal is up. The precise measurements of rotation angles were performed with the “angle” option in Photoshop or Image J. Note the regular and largely synchronous rotation throughout the process.

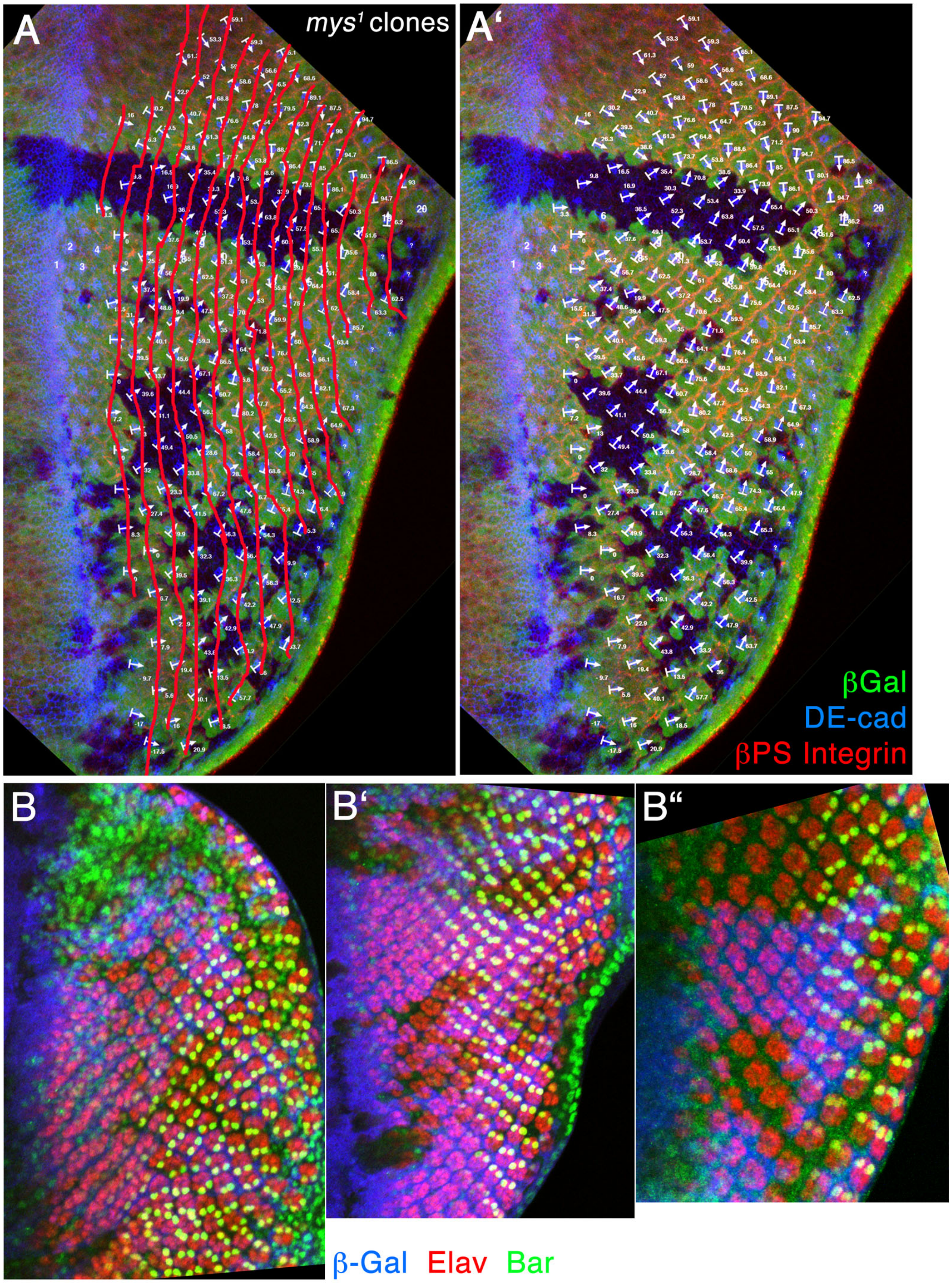

Figure S2: Rotation angle evaluation in βPS/mys mutant tissue

Eye imaginal discs from 3rd instar larvae containing *mys*- mutant tissue analyzed for the rotation angle distribution of each precluster. βGal staining marks wild-type tissue (shown in blue) surrounding the

mutant *mys*- clones. Anterior is to the left and dorsal is up. The analysis of rotation angles was performed using the “angle” option in Adobe Photoshop, with the individual values exported into Excel files and then analyzed with the RoseNet software statistically and graphically.

(A-A’) Staining for DE-cad allows the evaluation of angles from the earliest stages of rotation (column 5 onward). Angles are indicated in white and columns are also numbered in white. The extent of the individual columns is marked by red lines in A. (B-B”) Examples of *mys* mosaic eye discs stained for Elav (blue) and Bar (green), to outline the ommatidial positions in clusters posterior to the 10th column.

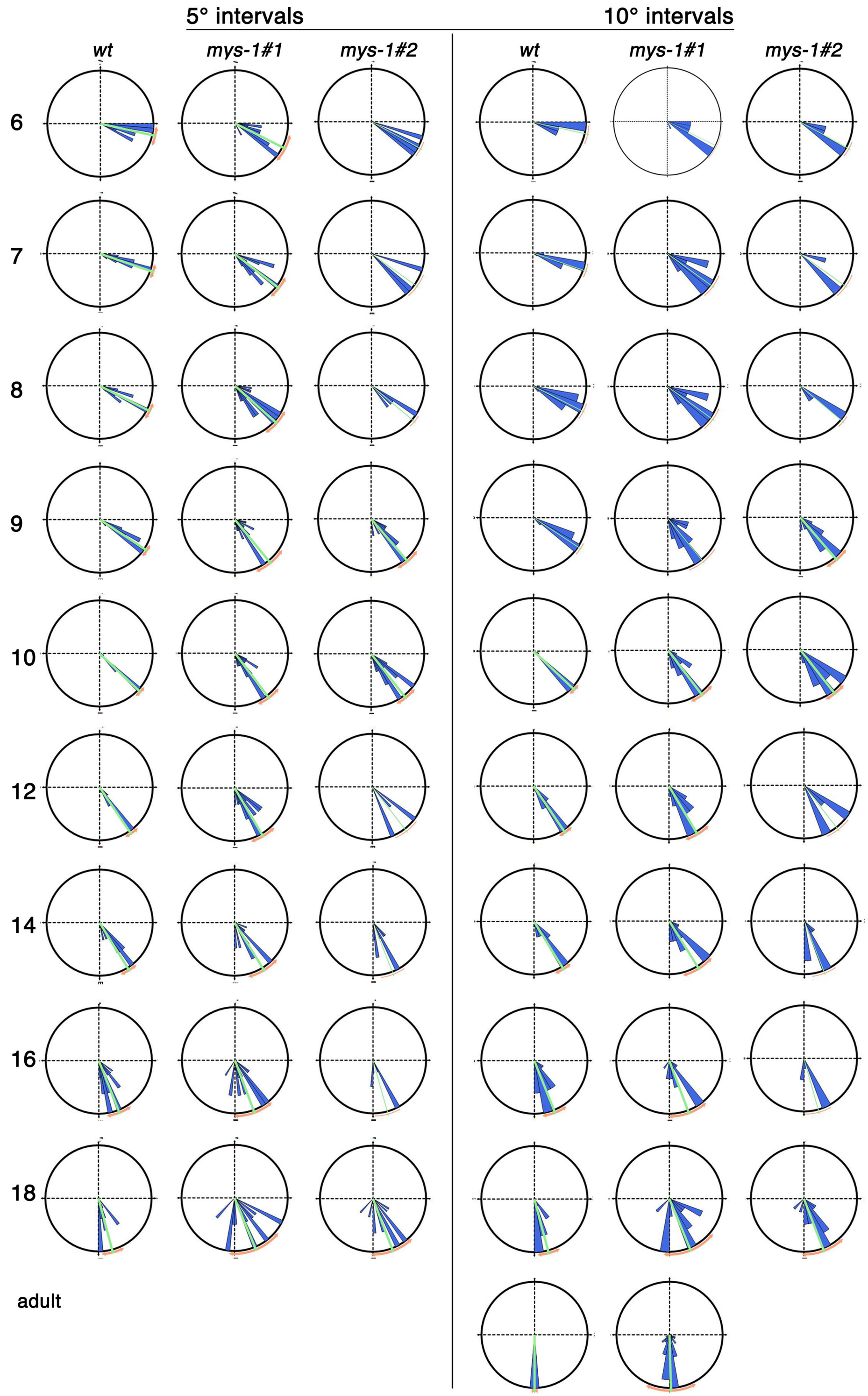
5° intervals
10° intervals
wt
mys-1#1
mys-1#2
wt
mys-1#1
mys-1#2
6
7
8
9
10
12
14
16
18
adult

Figure S3: Additional data set of numerical angle distribution evaluation and comparisons
Compilation of data (from examples shown in main figures and additional data sets). The initial measurements of rotation angles were performed with the “angle” option in Adobe Photoshop or Image J. Individual values were exported into MS Excel and aligned there from individual discs. These were then analyzed with the RoseNet software statistically and graphically as shown here. The angle distribution is presented either in 5° intervals (left side of figure) or in 10° intervals (right side). The 0° angle is to the right and 90° is down in all graphs. The column numbers are indicated on the left side. The mean rotation angle is shown as a green line and the standard deviation by an orange line along the periphery of the circle. Note that individual examples of *mys* mutant tissue always display a much larger angle distribution at any stage analyzed, but show comparable values between themselves for the respective stages. Note also that the variation/extent or distribution of angles in *mys* mutant vs. wild-type tissue is not affected by the size of the sectors used to display this: e.g. a display of 5° or 10° wedges does not affect the conclusions and further documents the reproducibility of the *mys* rotation defects

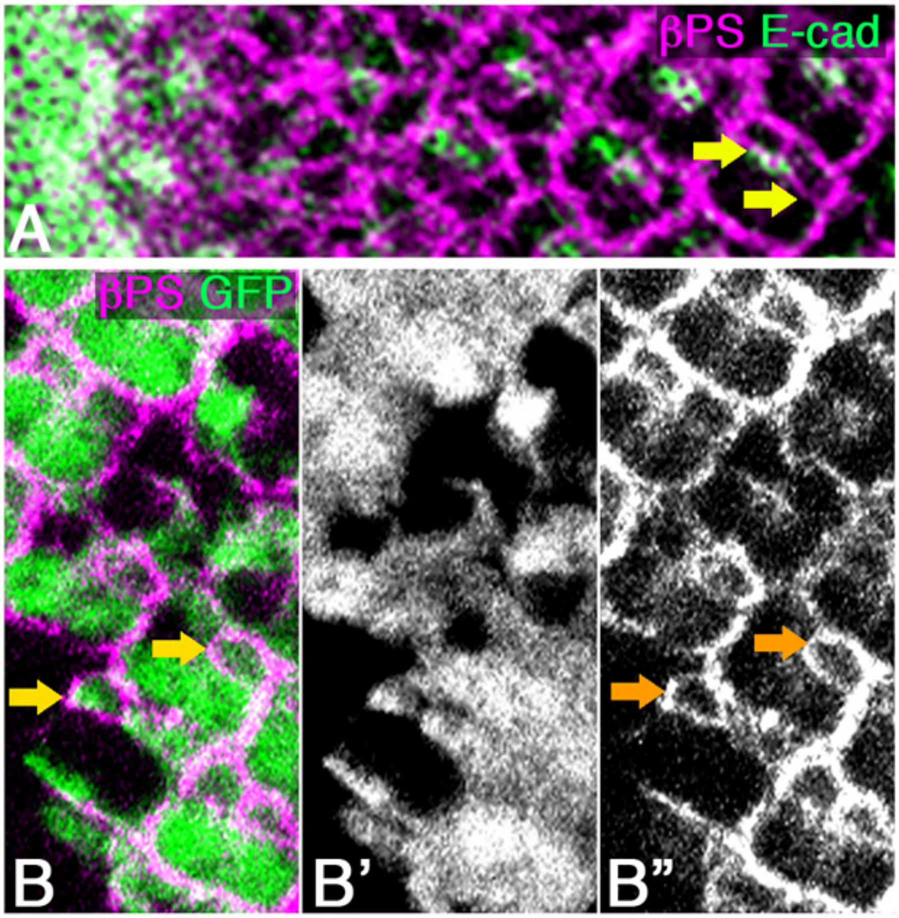

Figure S4
βPS/Mys is also enriched in developing R1/R6 (examples highlighted by yellow and orange arrows) at a time when the R7 precursor is being established. This R1/R6 specific enrichment persisted for approximately 2-3 columns. It is possible that the frequent loss of R7 observed in the dominant negative *sev>mys* background (main text Fig. 4B,D and Fig. 7A-B) is caused by a specific βPS/mys-integrin requirement in R1/R6, as R7 is being recruited at the time of integrin enrichment in R1/R6. However, this observation is outside the scope of this study and will need to be confirmed.
The enrichment in R1 or R6 is cell-autonomous, and lost from single mutant R1 or R6 cells, if either is mutant (orange arrows in panel B”, respectively), as observed in mosaic ommatidial clones.

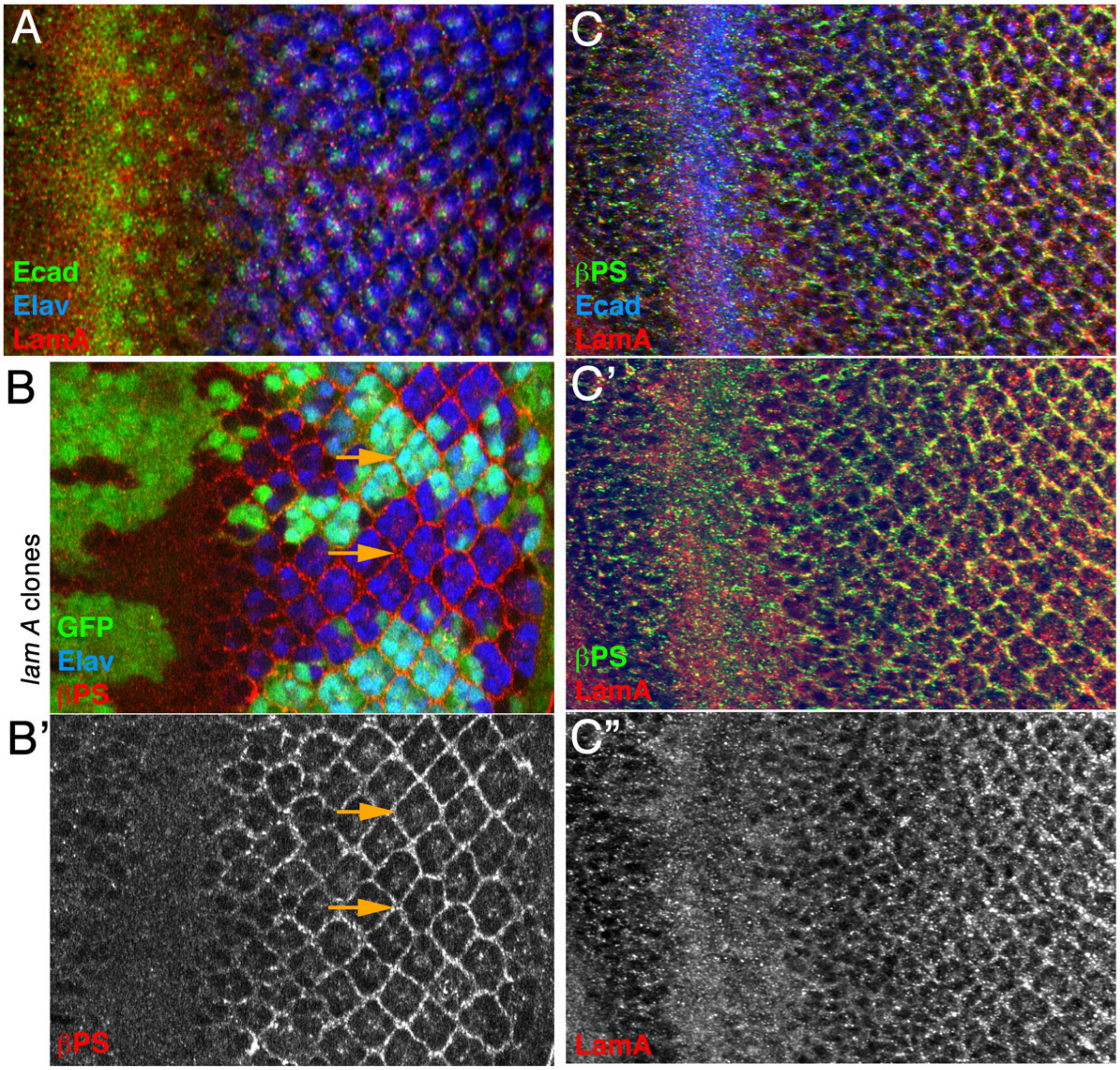

Figure S5. LamininA expression and localization in developing eye discs
Eye imaginal discs stained for LamininALanA and other markers as indicated. The MF is always towards the left side, and orientation is anterior to the left and dorsal is up. The general eye disc differentiation pattern is marked in blue either by Elav (**A** and **B**) or E-cad (**C**). Note that LanA is generally detected between the preclusters and maturing clusters in more posterior regions (right) of the discs.
Mutant clones of LanA (marked by absence of GFP in **B**) do not affect localization of integrins, see example for βPS/Mys (red in **B** and monochrome in **B'**). Orange arrows highlight clusters in a wild-type and mutant region (**B-B'**), revealing no detectable change in βPS/Mys levels or localization.
C-C": Co-staining of βPS/Mys (green) and LanA (red) reveals that in posterior (right) area of eye discs, they localize to a similar partially overlapping patterns (E-cad is in blue in **C**).
